# From a Pathophysiological Concept to a New Drug

**DOI:** 10.1192/j.eurpsy.2022.116

**Published:** 2022-09-01

**Authors:** G. Németh

**Affiliations:** Gedeon Richter Plc, Neuropsychiatry Global Portfolio, Budapest, Hungary

**Keywords:** pathophysiology, Antipsychotics, Clinical research, Dopamine

## Abstract

Although antipsychotics were discovered over fifty years ago, it took another decade until dopamine antagonism was demonstrated as central to their clinical effectiveness. Since accumulated evidence implicates the dopamine system in the pathophysiology of schizophrenia, all licensed first-line treatments operate primarily via antagonism of the dopamine D2 receptor. However, dopamine D2 receptor blockade does not effectively treat negative, cognitive and affective symptoms and, in a significant proportion of patients, it does not improve positive symptoms either. Therefore, additional neurochemical targets were considered. The “revised dopamine hypothesis” proposes that positive symptoms emerge due to hyperactive dopamine transmission in mesolimbic areas, while hypoactive dopamine transmission via the mesocortical pathway in the prefrontal cortex is linked to negative, cognitive, and partly affective symptoms. In this context, the role of D3 receptors were recognised. However, there is also evidence for the involvement of other neurotransmitter systems, suggesting that dopamine signalling relies on a suite of receptors that are thought to either facilitate or inhibit neurotransmitter activity through several interconnected neural circuits. Furthermore, there seem to be clusters of symptoms that cross the boundaries of disorders. Symptoms having similar pathophysiology at neurotransmitter level can be treated with the same drug or class of drugs. Thus, one particular drug might be effective in more than one indication. This lecture aims to illustrate the process of a new drug development by explaining how the underlying pathophysiology on receptor level impacts clinical studies and vice versa.

**Disclosure:**

Employee of Gedeon Richter Plc.

